# Moisture dynamics and ageing in clarinet reeds by neutron radiography

**DOI:** 10.1371/journal.pone.0334660

**Published:** 2025-12-03

**Authors:** Jongmin Lee, Qianru Zhan, Pavel Trtik, David Mannes

**Affiliations:** 1 PSI Center for Neutron and Muon Sciences, Villigen PSI, Switzerland; 2 PSI Center for Energy and Environmental Sciences, Villigen PSI, Switzerland; Charles University: Univerzita Karlova, CZECHIA

## Abstract

The vibrational behavior of reeds in wind instruments is highly sensitive to their moisture content, yet the underlying hydration dynamics and their relationship to ageing remain poorly understood. In this study, we employ in situ neutron radiography to visualize and quantify internal water distribution in clarinet reeds at three stages of use: pristine, broken-in, and aged. Reeds were subjected to controlled wetting durations followed by drying under ambient conditions to enable time-resolved analysis of moisture uptake and retention. Neutron imaging revealed that water preferentially accumulates in the vamp region due to longitudinally exposed vascular structures, while the stock remains largely dry. Ageing significantly reduces both water uptake and retention capacity. Pristine reeds absorbed water rapidly and uniformly, whereas aged reeds exhibited faster drying; asymmetric moisture distribution; and non-uniform swelling and shrinkage, consistent with structural fatigue. These results demonstrate how repeated use alters hydration behavior and underscore the governing role of anatomical features, such as exposed vascular bundles and parenchyma cells. This study presents the first application of neutron imaging to clarinet reeds, providing spatially resolved insight into internal moisture dynamics. The findings establish a scientific basis for understanding reed break-in and ageing, with implications for performance optimization, reed design, and maintenance practices. Moreover, this work pioneers a novel application of neutron imaging in the study of woodwind instruments, where moisture control critically influences playability and longevity.

## Introduction

The modern clarinet originated from the chalumeau (the name of lowest register of clarinet) that describes a pipe instrument with a single or double reed [1]. Bore, tone hole designs, and key mechanisms of the clarinet have been significantly improved, enhancing playability, intonation, and timbre consistency over the last two centuries. The sound of the clarinet starts from a vibrating single reed placed on a mouthpiece. As the player blows air through a gap between the tip of the mouthpiece and the reed, the reed vibrates, modulating the gap and projecting a resonance into the cylindrical bore [2]. One of the unique characteristics of single-reed instruments is a closed air column (only one end is open), rendered by a reed acting as a pressure control air valve [3]. Unlike open column instruments (both end open) such as the flute, where harmonics consists of octaves, the clarinet produces harmonics at odd-numbered intervals, often referred to as overtones [4]. The resonance frequency of the reed is in the range of 2–3 kHz, which can be varied by reed dimensions, moisture content, and stiffness [5]. The properties and quality of reeds have crucial influences on sound response, pitch, and air resistance [6]; in fact the sound not only start from the reed but also end on the reed.

**Fig 1:**
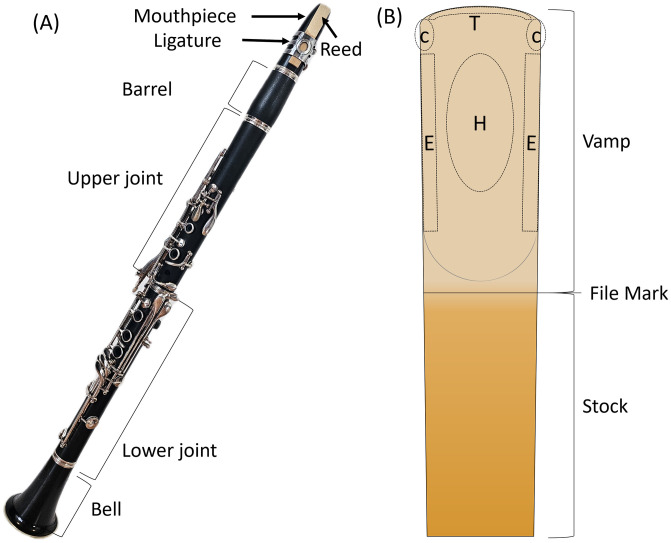
Anatomy of a clarinet and reed. (A) Photograph of a clarinet with labeled components. The mouthpiece assembly is rotated for clarity. (B) Reed schematic indicating key regions: tip (T), corners (C), edge (E), and heart (H). The epidermis of Arundo donax is retained on the stock but removed from the vamp. The vamp thickness gradually tapers from the file mark toward the tip.

Single reeds for the clarinets are made from the wood of the giant reed grass, *Arundo donax*, originated from the Mediterranean [7]. After drying step (up to several years) to remove moisture, the cane tube (raw, cylindrical stalk of Arundo donax) is split into blanks, in which the inner-tube side is flattened [8]. Then, they are machined to have a tapered tip to be placed on a tip of a mouthpiece. The dimensions of a single reed depend on the instrument and size. Its general anatomy is provided in Fig 1.

The vibration and playability of the reeds are heavily influenced by hydration level. Dry reeds are stiff and brittle, making it nearly impossible to initiate and sustain vibration. Moisture softens cellulose components of Arundo donax, increasing flexibility and reducing stiffness (Young’s modulus) [9], leading to better sealing between the tips of the mouthpiece and reed, which enables sound production. Several theoretical and experimental studies have been conducted to investigate the behavior of single reeds in the air column, by employing acoustic models [2–4] and two-dimensional (2D) simulations [10–13]. Gaillard et al. [14] provided a comprehensive review of reed mechanics, highlighting both the elastic properties of *Arundo donax* and the interactions involved in sound generation. Despite these efforts, the role of moisture in reed performance has not been yet fully characterized. In particular, the moisture content, its spatial distribution across the vamp and heart regions, and the wetting and drying behaviors are typically considered to be uniform. This heterogeneous moisture distribution is critical to understanding of breaking-in of pristine reeds [15,16], degradation of reed by saliva [17], and mechanical deformation under hygroscopic cycles [18].

To address this gap, a non-destructive diagnostic technique, capable of resolving internal moisture dynamics in real-time, is needed. Neutron beam is a unique probe, often considered as the complementary to X-ray. While X-ray interacts with electron clouds in an atom, neutron does with the nucleus. Therefore, neutron beam is highly sensitive to hydrogen containing elements compared to metals. Among various neutron techniques (e.g., diffraction, scattering, and spectroscopy), imaging has been successfully applied to samples requiring non-destructive diagnostic, including construction materials, nuclear materials, electrochemical devices, cultural heritage, and geoscience [19]. Recently, Lehmann et al. [20] outlined neutron imaging application to investigate historical musical instruments. Mannes et al. [21] showed three-dimensional (3D) neutron tomography of an entire trumpet. In the subsequent work, Streiger et al. [22] compared tomograms of a cylinder section of brass instrument at the beginning and end of the playing periods, in which the difference was attributed to deformation due to playing or corrosion. Further research involving neutron imaging and musical instrument focused on quantifying water content in an instrument in a whole or in part. Lehmann et al. [20] revealed moisture distribution inside cornet induced during playing. Lammlein et al. [23] quantified water level in tarnished violin wood under varying relative humidity (RH) levels coupled with frequency responses with varying layers of tarnish. To the best knowledge of authors, there has not been a research work investigating a woodwind instrument using neutron imaging, although it can deliver scientific insight to instrument design and development, considering hygroscopic cycles resulting in hydration and dehydration of main body (typically blackwood) and reeds.

In this work, clarinet reeds at different stages of their life cycle, including pristine, broken-in, and aged conditions, are investigated using in situ neutron radiography. To spatially resolve internal moisture dynamics, the reeds were imaged during drying under ambient conditions followed by controlled wetting durations. Real-time analysis of water distribution revealed how usage-induced degradation affects moisture uptake and drying behavior. By integrating in situ neutron imaging with knowledge of the anatomical structure and material response of *Arundo donax*, this study advances the quantitative understanding of moisture behavior in single reeds and its implications for vibrational performance and life cycle management. To the best knowledge of the authors, this work represents the first study applying neutron imaging to clarinet reeds, and more broadly to single-reed instruments, thereby establishing a novel bridge between neutron diagnostics and musical instrument research.

## Methods

### Description of reeds samples

Nine commercial clarinet reeds, 3.5 strength V12^®^ (Vandoren, Paris), were studied at varying stages of life-cycle: beginning-of-life (BoL), broken-in, and end-of-life (EoL). Hereafter, the broken-in reeds are referred as middle-of-life (MoL) for consistency with other two terms. For the BoL samples (annotated as Reed 1, 2, and 3), the original packaging from the manufacturer was opened just before the experiment. The MoL reeds (Reed 4, 5, and 6) were played on a professional setup daily for 1–2 weeks with incremental playing time (e.g., under 1–2 min during the first week and 5 min or more during the second week). In the second week of breaking-in, two sides (Fig 1) and bottom face of reeds were adjusted slightly by rubbing them on an etched glass to improve the sound response. The EoL reeds (Reed 7, 8, and 9) were played excessively (> 20 hrs) over several months after the break-in process. The summary of the studied reeds is shown in Table 1. Hereafter, each reed is identified by R followed by the number, i.e., R1 for Reed 1. Between playing sessions of MoL and EoL reeds, they were stored in a dedicated case under ambient temperature and humidity after excessive moisture removal. The case consisted of a glass panel bottom and fabric top to apply gentle pressure on the tip of the reeds to prevent warping.

**Table 1:** Summary of tested reeds under varying wetting time.

Wetting time (s)	Beginning-of-Life (BoL)			Middle-of-life (MoL)			End-of-life (EoL)		
	Reed 1	Reed 2	Reed 3	Reed 4	Reed 5	Reed 6	Reed 7	Reed 8	Reed 9
5	–	–	–	✔	–	–	–	–	–
120	(✔)	✔	✔	✔	✔	✔	✔	✔	✔
2400	✔	–	–	✔	–	–	✔	–	–

Check marks indicate studied conditions for which neutron imaging was conducted. For Reed 1 with 120s wetting was imaged; however, sample displacement during imaging limited quantitative analysis. The shading in cells represents a group where reeds were investigated simultaneously via neutron imaging.

### Sample preparation: Wetting and placing reeds on the stage

**Fig 2:**
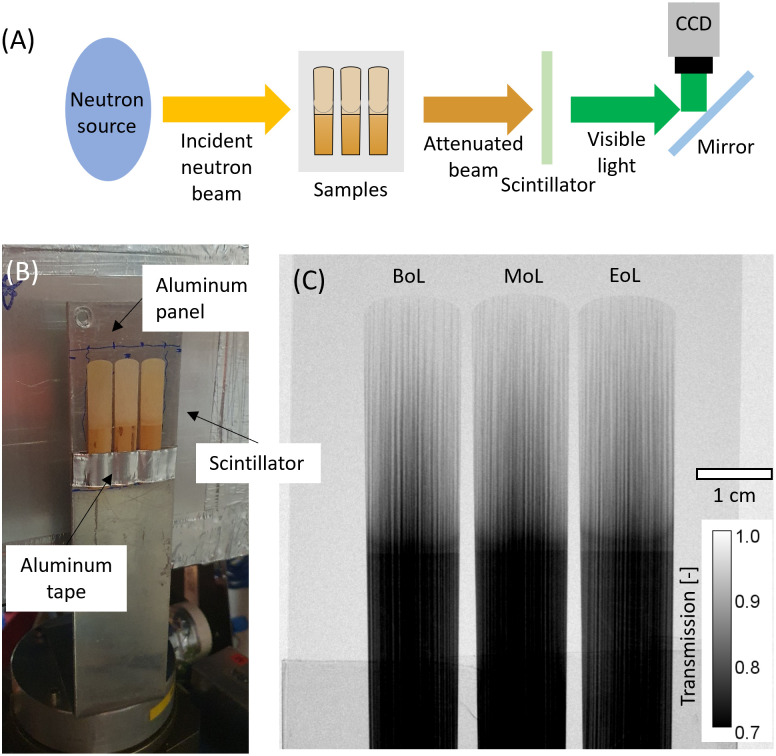
Neutron imaging setup and examples. (A) Schematic of neutron imaging test arrangement. CCD: charge-coupled device. (B) photograph of the sample setup at the NEUTRA beamline. (C) example transmission image of dried reeds (fully processed).

To study hydrodynamic behavior in a realistic condition, it is desirable to install complete clarinet setup in the neutron imaging stage. However, due to space constrain and issues with sample radioactivation with neutrons, only reeds were investigated in this study. The reed-mouthpiece combination was tested (S1 Fig); however, the composition of the mouthpiece (ebonite) led to strong neutron attenuation, limiting information on the reeds. The setup is shown in Fig 2B, where a 2 mm aluminum sheet, which is nearly transparent to neutron beam, was used to hold three reeds at three lifetime stage: R1, R4, and R7; R2, R5, and R8; and R3, R6, and R9, with an exception of R4 for 5s which was tested alone as a feasibility demonstration.

Prior to neutron imaging, reeds were completely submerged in de-ionized water under ambient conditions for 5, 120, and 2400 s to vary moisture levels. After taking the reed out of water, excess water on the surface was removed carefully, and reeds were placed immediately on the aluminum sheet in the imaging stage. The imaging last for 1 hour during drying. Preliminary on-site image processing was performed to verify dryness of the reed (i.e., there is no more intensity change over time).

A limitation of this method was the 2–3 minute gap between sample preparation and the start of neutron imaging, caused by the hutch exit protocol and opening of multiple beam shutters. Assuming linear drying behavior, the initial water uptake was estimated by extrapolating between the start of imaging and the point of complete drying. It was roughly estimate that 5–60% of water evaporated during the first 3 minutes without imaging, depending on the initial water content—for example, ~ 60% loss in the tip region of an aged reed after 5 s wetting, compared to ~5% loss in the heart region of a pristine reed after 2400 s wetting. For this reason, the results and discussion focus on reeds wetted for 120 s and 2400 s. In future studies, maintaining a controlled relative humidity environment could minimize premature drying prior to imaging; alternatively, a motorized axis could be implemented to automate reed wetting remotely.

### Neutron imaging

Time-series neutron imaging was performed at the NEUtron Transmission RAdiography (NEUTRA) beamline [24] of the Swiss Spallation Neutron Source (SINQ) [25]. This thermal neutron imaging beamline is characterized with a neutron flux of 9.8E6 [neutron/cm^2^/sec/mA] (SINQ operates around at 2mA), which has been used for studying cultural heritage and brass instruments mentioned earlier [20]. Fig 2A illustrates the working principles of neutron imaging. The attenuated beam through a sample is captured by a 30-µm -thick Gd_2_O_2_S -based scintillator screen that produces visible light, the intensity of which is based on the number of captured neutrons. Finally, Andor iKon-L charge-couple detector (Oxford Instruments) was used to record the visible light on the scintillator. The physical dimension of the sensor is 27.6 × 27.6 mm^2^ (2048 × 2048 pixels, 13.5 µm pixel size); therefore, a macro lens was used to fix the field-of-view (FoV) to 68.6 × 68.6 mm^2^, and the resulting pixel size was 33.5 µm at the acquisition rate of 10 s per frame. The imaging was performed for 5 times for the combinations presented in Table 1. For 2-min and 40-min wetted samples, the imaging was acquired for 360 frames (~1 hr), whereas for 5s wetted R4, imaging duration was 15 min. In all cases, the average of last 10 images represents a dry (reference) image. Additionally, dark current (DC), and open beam (OB) images were also recorded for image treatment. The raw images have been uploaded on the Zenodo database (10.5281/zenodo.17219051). The processed images as well as the Jupyter Notebook used to treat raw images are available upon request from the authors.

### Image processing

**Fig 3:**
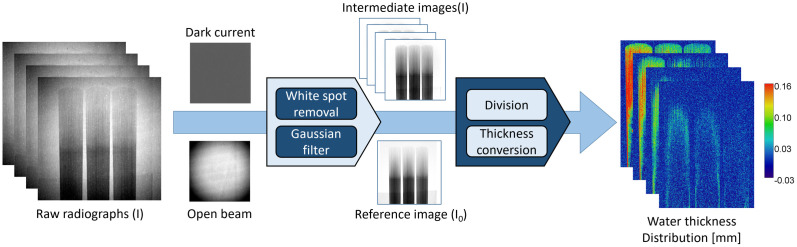
Flow chart summarizing image processing steps from raw image to water thickness map.

An open-source Python program [26] was used to process the acquired 16-bit raw radiographs and to extract water distribution from background signals. As seen in Fig 3, the processes include three-dimensional (3D) median filter to replace white spots caused by gamma rays; DC subtraction to account for camera background noise (measured without beam); OB normalization to correct for beam geometry; image registration and dose corrections; and dry frame referencing to extract water contents from reeds followed by thickness conversion. Representative raw, intermediate, and final processed images are provided in Section 5 of the Supplementary Information (S7 Fig).

The principle of material quantification based on beam attenuation through a sample is described by the Beer-Lambert:


$$I=I_{\text{0}}\times e^{-\sigma_{T}\delta_{N}t},$$ (1)

where $I_{\text{0}}$ is the incident beam before interacting with a sample, I is the attenuated beam through the sample, $\sigma_{T}$ is the neutron cross-section, $\delta_{N}$ is the atomic density, and t is the thickness of the sample. In general, the neutron cross-section and atomic density of the sample can be experimentally measured or obtained from a reference source. Their product defines the material attenuation coefficient, which characterizes how strongly the material attenuates the neutron beam and allows the quantification of the sample in terms of thickness.

For the applications where sample physical changes occur during time-series imaging, the Beer-Lambert law can be directly applied to images where $I_{\text{0}}$ and I are substituted with images in the reference (dry) state and drying state, respectively. In this study, the water attenuation coefficient of 0.35 mm^-1^ was used, which is specific to the NEUTRA beamline [27], For the reference image, last 10 images in each time-series acquisition were merged. To improve statistics for in situ images, 360 frames were binned into 72 images, taking average of every 5 frames. To perform consistent quantitative analysis among samples, it is necessary to compare the same region-of-interests (ROIs) across reeds. To achieve this purpose, individual reeds were cropped from processed images (containing three reeds except 5 s-wetting) and aligned to the same position manually by referencing the vertical position of file mark and coordinates of two corners.

### Image analysis

**Fig 4:**
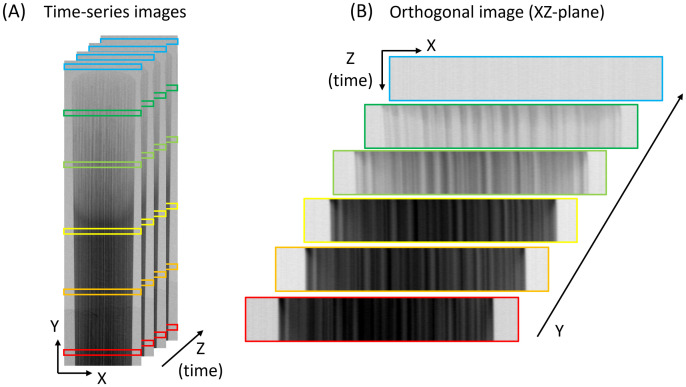
Orthogonal resplicing of time-series images. (A) Time-series transmission image of a reed in XY-plane (horizontal-vertical, space domain) in Z-axis (time). (B) Orthogonal images of (A) in XZ-plane (horizontal-time, space-time domain) at Y-axis (vertical) positions averaged annotated in colored boxes.

Three types of analyses were performed for each reed: plotting horizontal profile, averaging for an area, and averaging orthogonal slices. Average water content was calculated by averaging pixel-wise water thickness values within the defined ROI on ImageJ or Python. A horizontal profile computes an average of all vertical pixels (in Y-axis) for the given X-position, which was used to study a dry reed structure and water distribution (Fig 9). To represent water content over time (as shown in the vertical axis of Fig 10 and 11), mean values for the given ROIs (e.g., corners, heart, and edges) in Fig 1 were computed. Before computing an average, in-house developed algorithm was used to create a mask for the ROI is created to set values outside of the area into not-a-number (NaN). Lastly, averaged orthogonal slices were used to compare shrinkage behavior of the reeds under drying. As illustrated in Fig 4, the XY-plane images were resliced in XZ-plane, averaged in Y values using ImageJ [28]. The swell/shrinkage behavior can be characterized by tracking the edges of vascular bundles.

One of the challenges in comparing water distribution/quantity is to select consistent areas among reeds. Prior to performing quantitative analyses, each reed was cropped and aligned based on the file mark and left/right edge coordinates to ensure consistent ROI application, using SciPy package [29]. This enabled systematic comparison among reeds, because a single set of ROIs can be applied to all reeds.

## Results and discussion

### Reed morphology in literature and neutron images of dry reed

**Fig 5:**
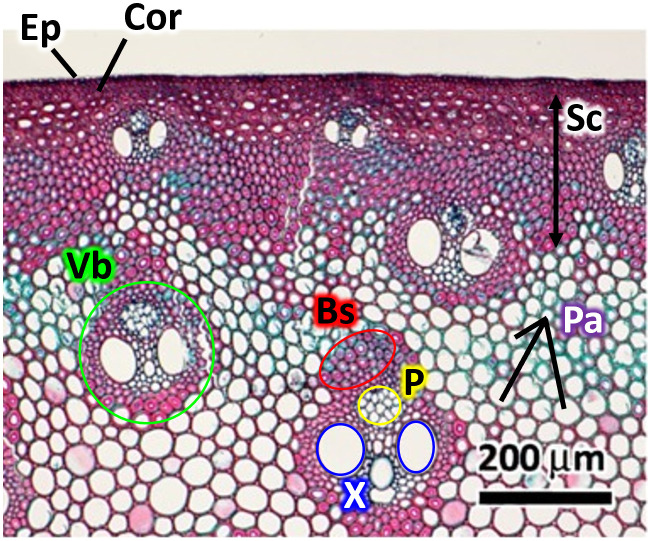
Light microscopy image of the thin traverse cross sections of a clarinet reed. Acronyms are as follows: epidermis (Ep), cortex (Cor), vascular bundle (Vb), vascular bundle sheath (Bs), sclerenchymatous cell (Sc), parenchyma cell (Pa), phloem (P), and xylem (X). Reproduced from Kawasaki et al. [30] with permission from John Wiley and Sons for publication in PLOS One.

The anatomical structure of *Arundo donax* reeds has been well-documented in prior studies, notably summarized by Gaillard et al. [14]. Kawasaki et al. [30] provided a detailed microstructural analysis using a variety of imaging techniques, including light, electron, and helium ion microscopy. As shown in Fig 5 (optical images reproduced from Kawasaki et al. [30]), the outermost layers of the reed consist of a monolayer epidermis, cortex, and sclerenchymatous cells, all of which are preserved in the stock portion of the reed. In contrast, the vamp region is machined to remove these outer layers, exposing the vascular bundles and parenchyma cells. Each vascular bundle comprises a tubular xylem paired with a phloem, embedded within a dense vascular bundle sheath. Parenchyma cells, characterized by thin cellulose-rich walls, function as water reservoirs and contribute to the reed mechanical damping properties.

**Fig 6:**
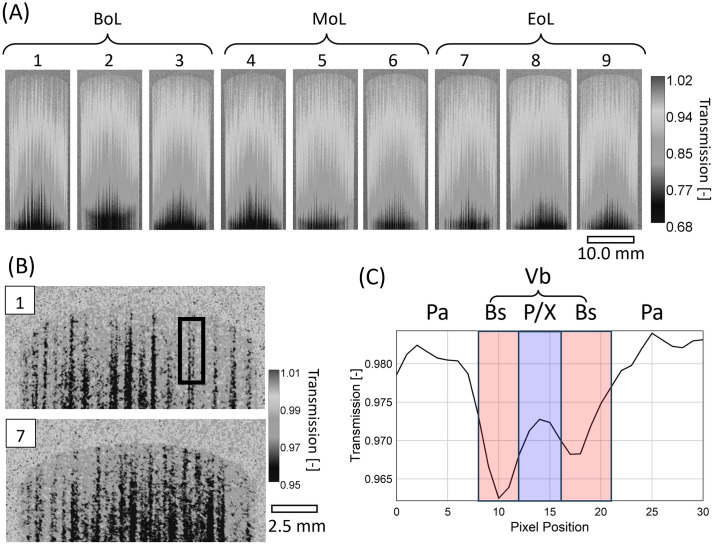
Neutron images (transmittance) of dry reeds and profile highlighting a vascular bundle. (A) Neutron transmission image of dry reeds: three BoL (1–3), three MoL (4–6), and three (EoL). Only vamp part of a reed is shown. (B) Enlarged images of the tip sections of Reed 1 and 7. (C) Horizontal profile of the highlighted region in the black box in (b), each pixel representing 33.5 µm.

Neutron radiographs of the vamp region for dried nine reeds (R1-R9) are shown in Fig 6A. The samples were imaged in a through-plane configuration, where the neutron beam passes orthogonally through the vamp thickness, integrating information along the beam path, as opposed to the optical microscopy that visualizes only the surface seen in Fig 5. As expected, transmission gradually decreases toward the vamp center due to increased material thickness. Overall, the external dimensions of BoL, MoL, and EoL reeds appeared consistent within the effective spatial resolution (~70 µm pixel size).

Detailed comparison of tip morphology between R1 (BoL) and R7 (EoL) reveals contrasting internal features, as shown in Fig 6B. In R1, vascular bundles exhibit a high-transmission central region, indicating a hollow or less dense core consistent with xylem and phloem tubes (Fig 6C). This observation aligns with the structure reported via microscopy, where dense sheaths encase fluid-conducting elements. Based on full-width-at-half-maximum (FWHM) analysis, the combined diameter of xylem and phloem was estimated at ~167.5 µm (5 pixels). In EoL reeds (R7–R9), however, such hollow structures were less distinct, suggesting partial collapse or filling of vascular elements over time.

Additionally, the MoL reeds (R4–R6), considered broken-in or acoustically optimal, displayed more symmetrical and uniform structural features in the tip and edge regions compared to BoL and EoL reeds. This observation supports previous findings on the functional optimization of reeds during early stages of usage [31]. Nevertheless, no definitive group-level differences were observed using neutron imaging alone, due to limitations in spatial resolution and signal-to-noise ratio. Recent advances in neutron imaging have demonstrated resolutions approaching 10 µm [32,33] by using advanced optics (such as magnifying lens and fibre optics taper) and thinner scintillators, however further spatial resolution improvement is hindered by limited neutron flux, even at the reactor-based neutron sources. In addition, high-resolution neutron imaging generally results in limited fields of view (<1 cm²) and prohibitively long exposure times, making them unsuitable for full-reed characterization.

### *In situ* water image in drying reeds

**Fig 7:**
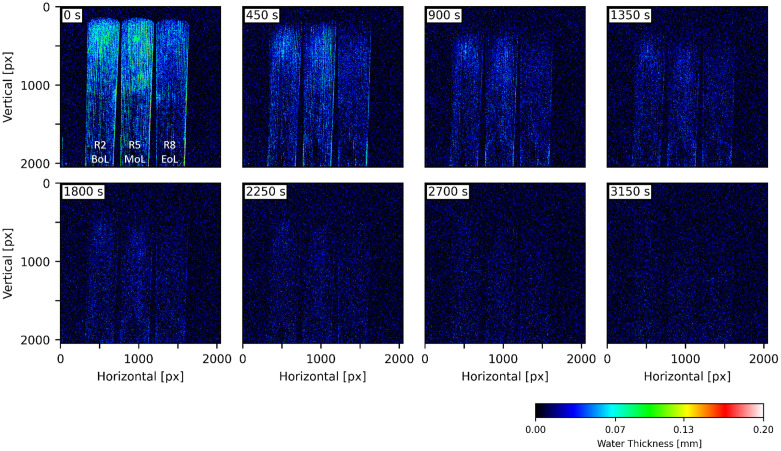
Water thickness images derived from in situ neutron radiography of 2-min-wet reeds drying. Three reeds are R2 (BoL), R5 (MoL), and R8 (EoL) from left to right in each timestamp. The reeds were immersed in water for 2 min before imaging.

A key advantage of neutron imaging is its high sensitivity to hydrogen, allowing precise mapping of water distribution in small quantities. Fig 7 illustrates the drying process of reeds R2 (BoL), R5 (MoL), and R8 (EoL) after 2 minutes of immersion. Among the three, R5 (MoL) exhibited the highest initial water content, followed by R2 and R8. The regional distribution in the vamp appeared relatively homogeneous along the vertical axis. The EoL reed consistently displayed lower water content in the tip and heart, indicating that extended usage impairs initial wetting capacity. Drying initiated at the tip region within 450 seconds, progressing toward the base. All samples were confirmed to be dry by 2250 seconds.

**Fig 8:**
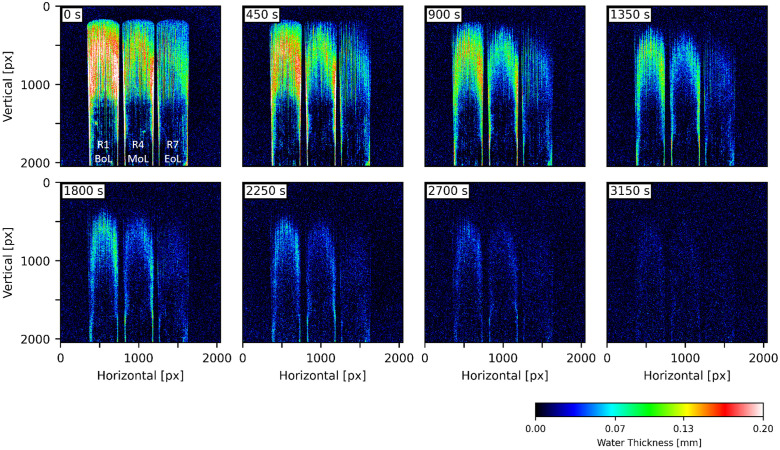
Water thickness images derived from in situ neutron radiography of 40-min-wet reeds drying. Three reeds are R1 (BoL), R4 (MoL), and R7 (EoL) from left to right in each timestamp. The reeds were immersed in water for 40 min before imaging.

A critical step in sample preparation, susceptible to experimental error, involved removing excess surface water from reeds following extraction from water. This was accomplished by gently wiping each reed with laboratory-grade wipes (Kimtech Science^TM^ Precision Wipes). Each reed was visually inspected to confirm the absence of residual water droplet and film prior to placing it on the aluminium sheet. However, neutron imaging can discern a small quantity of water thickness down to 10–20 µm, and any excess water may negatively affect data analysis. To mitigate this issue, reeds were immersed in water for longer duration (40 min), thereby increasing water uptake compared to the background noise signal and possible experimental error, as shown in Fig 8. Notably, the BoL reed exhibited a significantly higher water content, especially in the center of the vamp, compared to MoL and EoL reeds. The BoL also maintained a symmetric water distribution about the longitudinal axis throughout drying. In contrast, the EoL reed showed asymmetric distribution, with greater water retention on the right half. This asymmetry is likely caused by structural degradation from prolonged use and manual adjustments (e.g., sanding), which can alter geometry and block surface pores. Additionally, white-red regions at the left and right edges of the lower vamp and stock (seen in BoL and MoL reeds) correspond to water thickness above 0.2 mm. This effect can arise from either sample displacement or localized swelling, as demonstrated in S3 Fig. In this case, swelling is the likely cause. No swelling was detected in the EoL reed, further supporting the hypothesis of structural fatigue and reduced hygroscopic response with ageing. Additional water thickness images for other reeds (R2, R3, R5, R6, R8, and R8) are provided in S4–S6 Fig. Localized swelling is discussed in further detail in the subsequent section.

### Water transport and wetting mechanisms in a reed

Before proceeding with quantitative analysis of water content during drying affected by ageing effects, it is worthwhile to understand the wetting behavior and water transport pathways in reeds. *Arundo donax* is known for its rapid growth rate and high water demand [34]. In the living plant, vascular bundles function both as primary water conduits and mechanical support structures, encased in rigid sclerenchymatous sheaths. Surrounding these bundles, parenchyma cells provide flexibility and damping due to their thin lignified walls and water-filled interiors. Water is transported axially through xylem by capillary action and subsequently absorbed into surrounding parenchyma cells [35]. While the sclerenchymatous sheath presents a hydrophobic barrier limiting radial water movement [36,37], lateral transport is still possible through diffusion and capillarity in regions with less dense or discontinuous sheathing. However, once *Arundo donax* culms are processed into reeds and subjected to multi-year drying, structural integrity is preserved, but wetting behavior has not been thoroughly studied.

In situ neutron radiography (Fig 7–8) revealed that after immersion, water preferentially accumulates in the vamp region rather than the stock. This is expected, as outer impermeable layers, epidermis, cortex, and sclerenchyma, are removed during vamp machining, whereas they remain intact in the stock. Additionally, the tapered geometry of the vamp exposes longitudinal sections of xylems and parenchyma cells, facilitating water entry. Surprisingly, after 2400 s of soaking, no significant xylem filling was observed in the stock region of the BoL reed (R1) in Fig 8. Only minimal water content was observed near the file marks and lower end, indicating that uptake is largely driven by surface interaction; however, a contribution from capillary filling cannot be ruled out given the spatial resolution of our measurements. Two mechanisms may explain this: (1) machining damage may have closed the xylem ends, disabling capillarity, and (2) submersion of an entire reed in water where air may have trapped within the xylem, preventing water ingress due to opposing hydraulic fronts. These observations indicate that under the experimental conditions tested in this study, water uptake in reeds occurs primarily through xylem filling in longitudinally exposed vascular bundles and absorption by parenchyma cells. In future studies, tailored wetting protocols (e.g., high-resolution microscopy with one-end wetting using a dyed or contrast solution, vacuum-assisted soaking, or pressure-controlled imbibition) are necessary to verify capillary contributions and to elucidate how structural damage and hydraulic boundary conditions influence water transport in reeds during different stages of use.

### Wetting duration effect on local water contents and drying behavior

**Fig 9:**
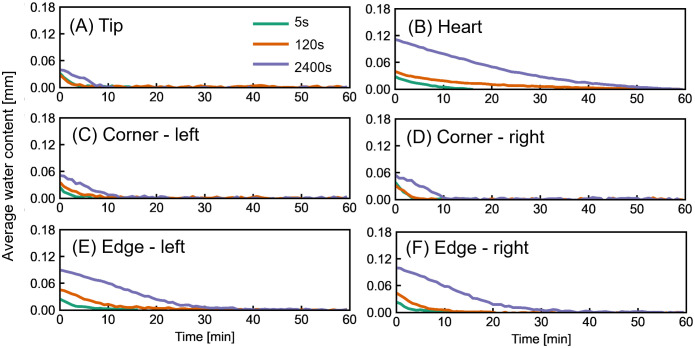
Effect of wetting duration on drying in various reed regions. R4 (MoL) reed was wetted for 5, 120, and 2400 s prior to imaging. (A)-(F) show averaged water content during 1 hr of drying in the tip, heart, corners, and edges.

Fig 9 shows the drying behavior of reed regions after wetting durations of 5, 120, and 2400 s for a MoL reed (R4), which best reflects practical playing conditions. In the tip and corners (Fig 9A, C, and D), initial water contents do not show a strong correlation with increased wetting. The longitudinally cut xylems and parenchyma cells spontaneously absorb water and enable rapid saturation. The drying process in these areas follows a nearly linear trend and completes within about 5 minutes, suggesting that water evaporation is primarily governed by evaporation on the surface. Because the tip and corner regions are thin (ranging from 100 µm to a few hundred µm), evaporation only occurs on the surface, rather than in a bulk part. In such thin-film case as ascribed in [38], evaporation rate is determined by air convection, which can be expressed in linear relationship.

In contrast, the heart and edge regions (Fig. 9B, E–F) exhibit a clear dependence of water content on wetting duration. Water content increased with wetting time, particularly in the heart region, where the increased local thickness of a reed is directly proportional to water storage volume. Extended wetting of 2400 s allowed for water to enter the deeper part of the reed. The drying curves in these regions are distinctly non-linear, which is indicative of diffusion-limited evaporation. Once the surface layer begins to dry, the evaporation front shifts progressively into the interior, and the increasing diffusion path length slows the overall rate of drying. As demonstrated by Solbrig et al. [39], neutron imaging of water distribution in wood-based composites can be directly related to vapor transport dynamics. In particular, Mannes et al. [40] showed that drying behavior in wood materials follows Fickian diffusion, where the drying rate is governed by an advancing evaporation front and the progressive increase of the diffusion path length:


$$J=\;{-D}_{eff}\times \frac{dC}{dx}\;,$$ (2)

where J represents diffusive flux [mol·m^-2^·s^-1^], $D_{eff}$ indicates diffusion coefficient [m^2^·s^-1^], and $\frac{dC}{dx}$ is the concentration gradient [mol·m^-3^·m^-1^] over a distance of dx. The non-linear drying response further reflects the differing roles of free and bound water in ligno-cellulosic materials [41,42]. Free water, located mainly in larger pores such as vascular bundles and lumina, dominates the early drying stage, whereas bound water associated with the cell wall matrix governs the later stages through slower diffusion

### Water uptake and surface effects during ageing

**Fig 10:**
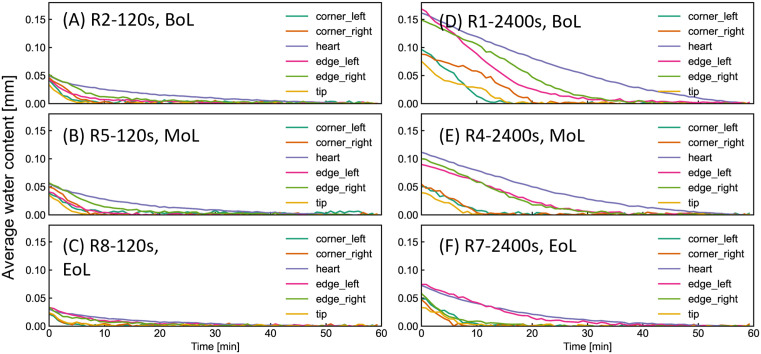
Averaged water content in corners, edges, heart, and tip. (A)-(C) corresponds to Reeds 2 (BoL), 5 (MoL), and 8 (EoL) after 2-min wetting shown in Fig 7. (D)-(F) are Reeds 1 (BoL), 4 (MoL), and 7 (EoL) after 40-min wetting in Fig 8. The same plots for all reeds are provided in S2 Fig.

To elucidate the influence of ageing on reed hydration behavior, average water content was quantified in selected ROIs across multiple reeds (Fig 10). Detailed data for all individual reeds are provided in S2 Fig. In the case of short-term wetting (120 s; (Fig 10A-C), BoL and MoL reeds exhibited similar levels of hydration, while EoL reeds showed a marked reduction, particularly in peripheral regions such as the edges and heart. Under prolonged immersion (2400 s; Fig 10D-F), ageing had a pronounced effect, with all ROIs in EoL reeds displaying diminished water uptake. The R1 data may contain error due to residual surface moisture not fully evaporated during the 1-hour drying period, particularly in the heart and corners. Nonetheless, comparison between R4 (BoL) and R7 (EoL) confirms that ageing leads to: (1) reduced water uptake and (2) diminished water retention capacity, evident in faster drying.

The degradation of reed structure through repeated playing arises from multiple factors: repeated vibration, mechanical wear, hygroscopic cycling (wetting with saliva or water followed by drying), thermal fluctuations (storage vs. warm breath), microbial decay from saliva, and the loss of soluble components such as sugars [43]. As noted by Rüggeberg et al. [44], the mechanical stiffness of vascular bundles significantly exceeds that of adjacent parenchyma tissue, making the latter more vulnerable to damage during use. Kemp and Scavone [45] employed in situ X-ray computed tomography to monitor reed microstructure under humidity cycling, reporting increased tortuosity and wall thinning in parenchyma cells, key indicators of fatigue and weakening. The subsequent optical microscopy work [46] confirmed the development of microcracks and structural deformation, contributing to elevated internal damping. More recently, Chen et al. [47] used optical coherence tomography to monitor reed degradation, showing reduced backscattered signal intensity associated with parenchyma cell deformation and evidence of vessel lumen expansion and bundle failure. The reduction in water uptake due to humidity cycling has also been documented in various materials. Esteban et al. [48] investigated hygroscopicity in wood under repeated sorption–desorption cycles and found that neutralization of OH groups in the cell wall reduced water-binding capacity, thereby lowering hygroscopic response. Similarly, Popineau et al. [49] demonstrated that repeated humidity cycling in composites decreases internal stresses due to microstructural damage, with the extent of degradation accelerated by both RH amplitude and cycle duration. Hygroscopic cycling also affects surface behavior. Kumar et al. [50] applied digital holographic interferometry to map surface deformation and strain fields, reporting that repeated wet–dry cycling produces non-uniform shrinkage once moisture levels fall below the fiber saturation point, reflecting heterogeneous surface moisture dynamics.

Another factor to consider is surface degradation that inhibits water penetration into the reed. For example, saliva in contact with dental biomaterials leads to the rapid formation of a salivary pellicle [51,52], suggesting that a similar thin, partially hydrophobic layer could form on reed surfaces, acting as a barrier to water uptake. Pore blockage by airborne particles, mineral impurities in water, and salivary deposits is highly plausible, resulting in reduced permeability and sorption capacity as well-known phenomenon across porous materials [53]. Surface wear is also relevant: studies on wood show that abrasion with sandpaper of increasing grit size (above 200) slightly increases hydrophobicity, as indicated by higher contact angles [54]. This effect is analogous to surface wear in reeds during playing and storage. Although these mechanisms have not yet been systematically characterized in clarinet reeds, they are widely recognized among musicians and reed manufacturers [55–58].

### Swelling analysis of reed between wet and dry

**Fig 11:**
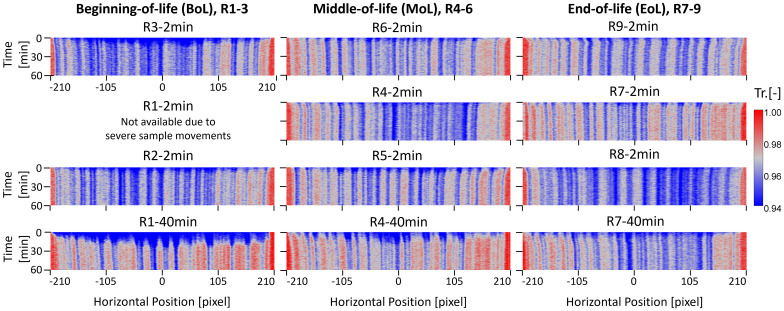
Orthogonal images in XZ-plane near the reed tip part of transmission images. The left, center, and right columns correspond to BoL, MoL, and EoL. A rectangular region with the height of 20 pixels, below two corners, were selected for averaging, represented as a single line in the time-axis. R1-2 min is not available due to severe sample displacement (see SI Section 5 and S3 Fig).

Another important indicator of reed ageing is dimension changes between wet and dry states, a phenomenon commonly known as warping that arises from inhomogeneous swelling/shrinking. Based on the wetting and drying dynamics observed, the tip region shows the highest sensitivity to water uptake and dimensional changes. Fig 11 presents neutron transmission images of reeds, resliced orthogonally in the XZ-plane, allowing direct visualization of shrinkage by tracking low-transmission regions (in blue) associated with vascular bundles.

In the BoL reeds, dimensional changes appear relatively uniform across the horizontal axis. Most shrinkage occurs within the first 5–10 minutes of drying, after which the spacing between vascular bundles, representing the intervening parenchyma cells, remains consistent, even after extended drying (e.g., R1–40 min). This suggests a reversible and symmetric swelling/shrinking cycle in BoL reeds. In contrast, MoL reeds begin to exhibit asymmetric behavior. For example, R5–2 min and R6–2 min demonstrate greater shrinkage on the right side of the tip compared to the left. This uneven contraction intensifies in EoL reeds, where localized, delayed, and chaotic dimensional changes become evident, suggesting microstructural fatigue and irreversible deformation. Such non-uniform shrinkage leads to tip warping and is consistent with parenchyma cell deformation observed in previous X-ray computed tomography [31,45,46] and optical coherent tomography [47], as discussed in the previous section. Because bound water is primarily associated with parenchyma cells and governs their swelling behavior [42], damage to these cells accelerates and unevenly amplifies shrinkage during drying. Overall, these findings indicate that while BoL reeds retain uniform swelling and drying behavior, this uniformity progressively deteriorates with age. Localized collapse and mechanical anisotropy introduced through repeated use ultimately compromise structural integrity of the reed.

To our knowledge, asymmetric shrinkage of a complete reed, particularly in relation to ageing, has not yet been explicitly investigated in the literature. Such non-uniform deformation is expected to alter vibrational response through asymmetric stiffness and geometry, and may also impair sealing against the mouthpiece, thereby disrupting the formation of a stable closed-column air cavity essential for sound production. Although acoustic measurements and simulations are beyond the scope of the present study, incorporating reed inhomogeneity into models [4] and experiments [59] represents a promising direction for future work.

### Perspectives on reed ageing and neutron imaging on woodwind instruments

*Arundo donax* has been studied for decades both as a biological species and as musical instrument reeds. However, a persistent gap remains between scientific insights and practical knowledge held by musicians. A clear example is reed ageing. While researchers have analyzed dried or rehydrated reeds to understand structural degradation, the findings often do not translate directly into terms meaningful for players. By integrating a common musician practice, reed soaking, we demonstrated how ageing influences water uptake and distribution in a clarinet reed. Our findings show that with prolonged use and storage, reeds gradually lose their ability to absorb and retain moisture. Aged reeds not only exhibit diminished mechanical compliance, but also struggle to maintain adequate internal hydration, a key factor underlying the loss of vibrational responsiveness reported by musicians. In contrast, pristine reeds absorb water rapidly and in excess, facilitating vibration but often requiring a break-in period. Interestingly, this break-in process, though it structurally damages internal tissues, may tune the reed toward a more playable and responsive state.

Beyond clarinet reeds, neutron imaging offers significant potential for both scientific and commercial applications in woodwind instrument research. Unlike string instruments, woodwinds are subject to continuous humidity fluctuations during play due to human exhalation and internal condensation. While surface condensation is typically removed after playing, internal moisture remains and dissipates gradually, contributing to dimensional changes in the instrument. In extreme conditions, such as exposure to cold air immediately after a performance, abrupt swelling and contraction can cause cracks in the instrument body, an issue well known to professional musicians. African blackwood, the standard material for clarinets and oboes, is highly valued for its density and acoustic properties but is increasingly rare and costly, being classified as an endangered species [60]. Understanding in situ moisture dynamics using non-invasive methods such as neutron imaging can aid in identifying alternative wood sources and in developing improved maintenance practices, potentially enhancing the longevity and sustainability of woodwind instruments.

## Conclusion

This study presents the first systematic investigation of water distribution and ageing effects in clarinet reeds using neutron radiography. By visualizing drying behaviors across reeds at different lifecycle stages, we provide critical insights into spatial distribution of moisture in reeds and how ageing alters water uptake, retention, and swelling/shrinkage. The main findings of this study are as follows.

- Water distribution follows a spatial and temporal gradient: In all reed stages, water accumulates preferentially in the vamp where surface machining enhances permeability, while the stock remains relatively dry due to intact epidermis and denser tissue layers. Water absorbance was attributed to surface wetting, rather than capillary in vascular bundles.
- Water uptake capacity declines with ageing: Beginning-of-life (BoL) and middle-of-life (MoL) reeds absorbed significantly more water than end-of-life (EoL) reeds, particularly in the vamp and heart regions.
- Ageing reduces water retention capability: Aged reeds exhibited accelerated drying behavior implying that water does not penetrate as deep as in fresh reeds.
- Swelling behavior becomes asymmetric with age: While BoL reeds showed uniform shrinkage during drying, MoL and EoL reeds displayed inhomogeneous deformation, which is linked to warping and acoustic instability.

These findings have implications not only for musicians and manufacturers seeking to improve reed performance but also for future studies exploring hydration behavior in other woodwind components and hygroscopic biomaterials.

## Supporting information

S1 Fig Reed on mouthpiece. (A) Neutron image of a clarinet mouthpiece with a moistened reed (120s fully immersed in water), normalized by open beam (transmission). (B) Vertical profile, top to bottom in the image, at the center of the mouthpiece. (C) Horizontal profiles on top, center, and bottom position in the baffle/window of the mouthpiece.
(TIF)

S2 Fig Average water content in various parts of reeds. Parts are corners, heart, edges, and tip. The title for each graph represents reed number (e.g., R7 corresponds to Reed 7) followed by wetting period. BoL: R1-3; MoL: R4-6; and EoL: R7-9.
(TIF)

S3 Fig Demonstration of reed displacement overtime during imaging. (A), (B), and (C): Transmission image (0, 250, 500, and 1000s from left to right), water thickness image, and horizontal profile corresponding to highlighted box in (A) of Reed 1 (BoL) after 2-min wetting. (D), (E), and (F): corresponding for Reed 2 (BoL) after 2-min wetting. The dotted lines in images indicate the edges of the reeds.
(TIF)

S4 Fig R4 drying after 5 s of wetting. (TIF)

S5 Fig R3, R6, and R9 drying after 120 s of wetting. (TIF)

S6 Fig R1, R4, and R7 drying after 120 s of wetting. (TIF)

S7 Fig Representative images of exp401 (R1, 4, and 7 with 40 min wetting) during image processing. (TIF)
