# Supplementary figures and images for: Moisture dynamics and ageing in clarinet reeds by neutron radiography
Source: PLoS One. 2025 Dec 3;20(12):e0334660. doi: 10.1371/journal.pone.0334660 (PMC12674550; doi:10.1371/journal.pone.0334660)

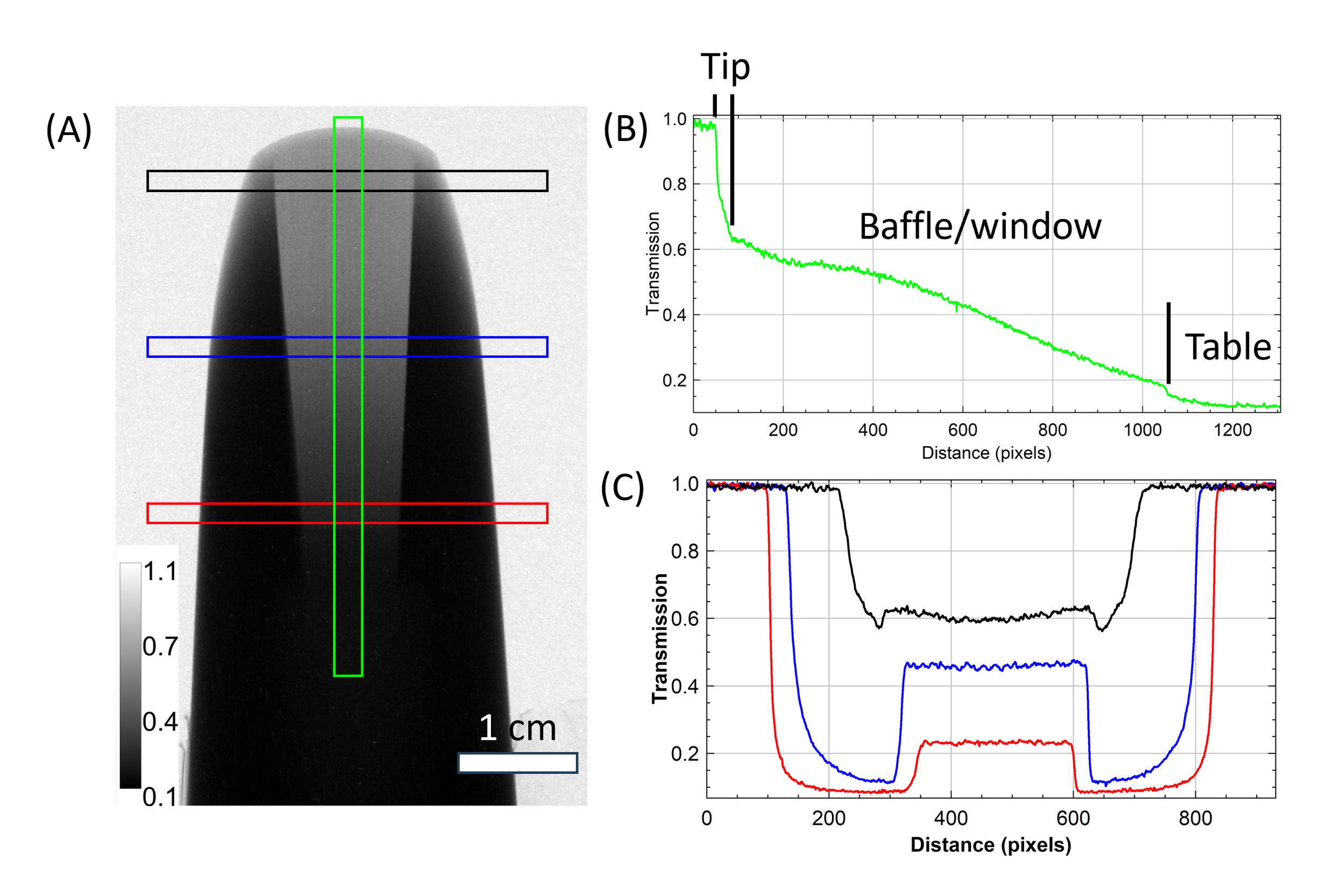

Supplement: S1 Fig — (A) Neutron image of a clarinet mouthpiece with a moistened reed (120s fully immersed in water), normalized by open beam (transmission). (B) Vertical profile, top to bottom in the image, at the center of the mouthpiece. (C) Horizontal profiles on top, center, and bottom position in the baffle/window of the mouthpiece. (TIF) [file pone.0334660.s001.tif]

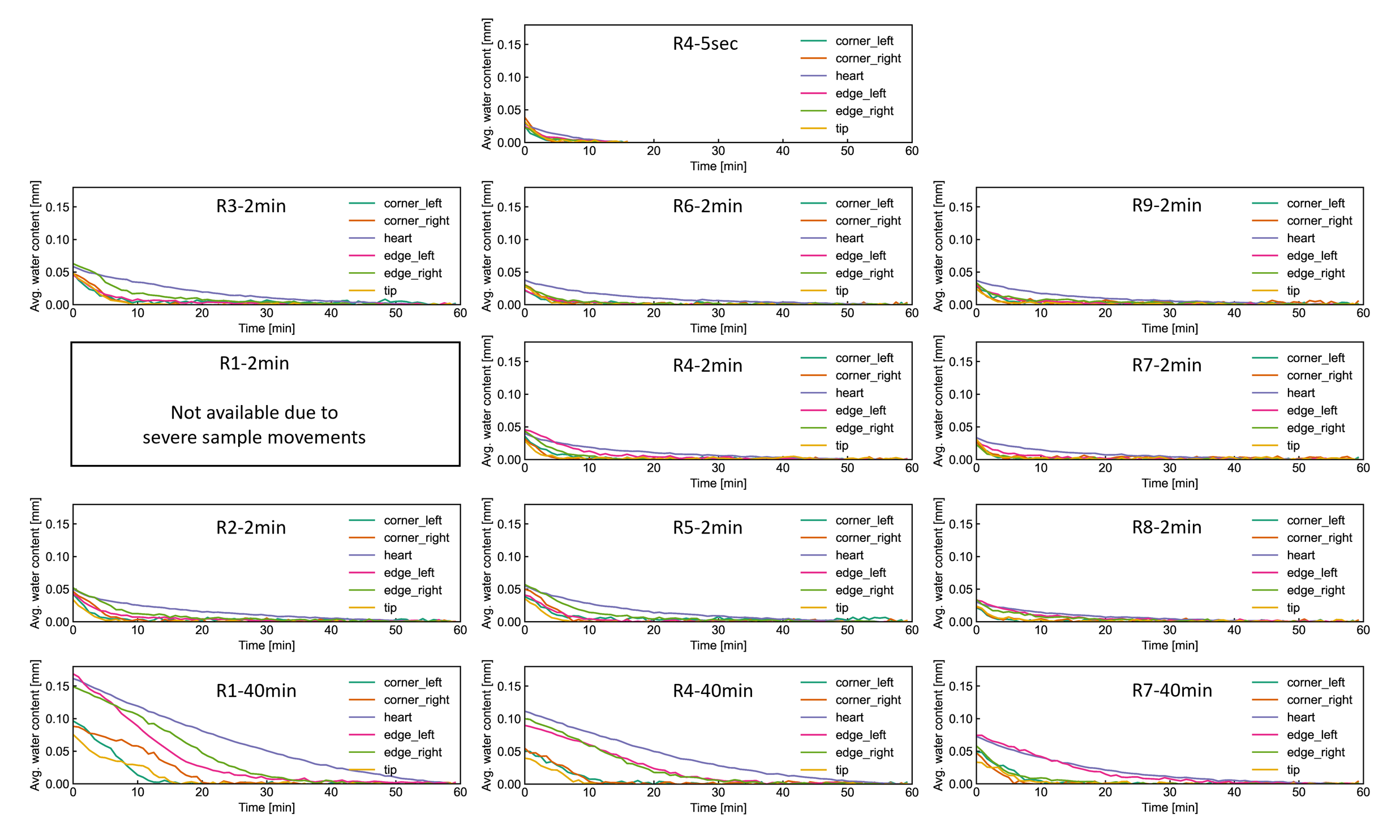

Supplement: S2 Fig — Parts are corners, heart, edges, and tip. The title for each graph represents reed number (e.g., R7 corresponds to Reed 7) followed by wetting period. BoL: R1-3; MoL: R4-6; and EoL: R7-9. (TIF) [file pone.0334660.s002.tif]

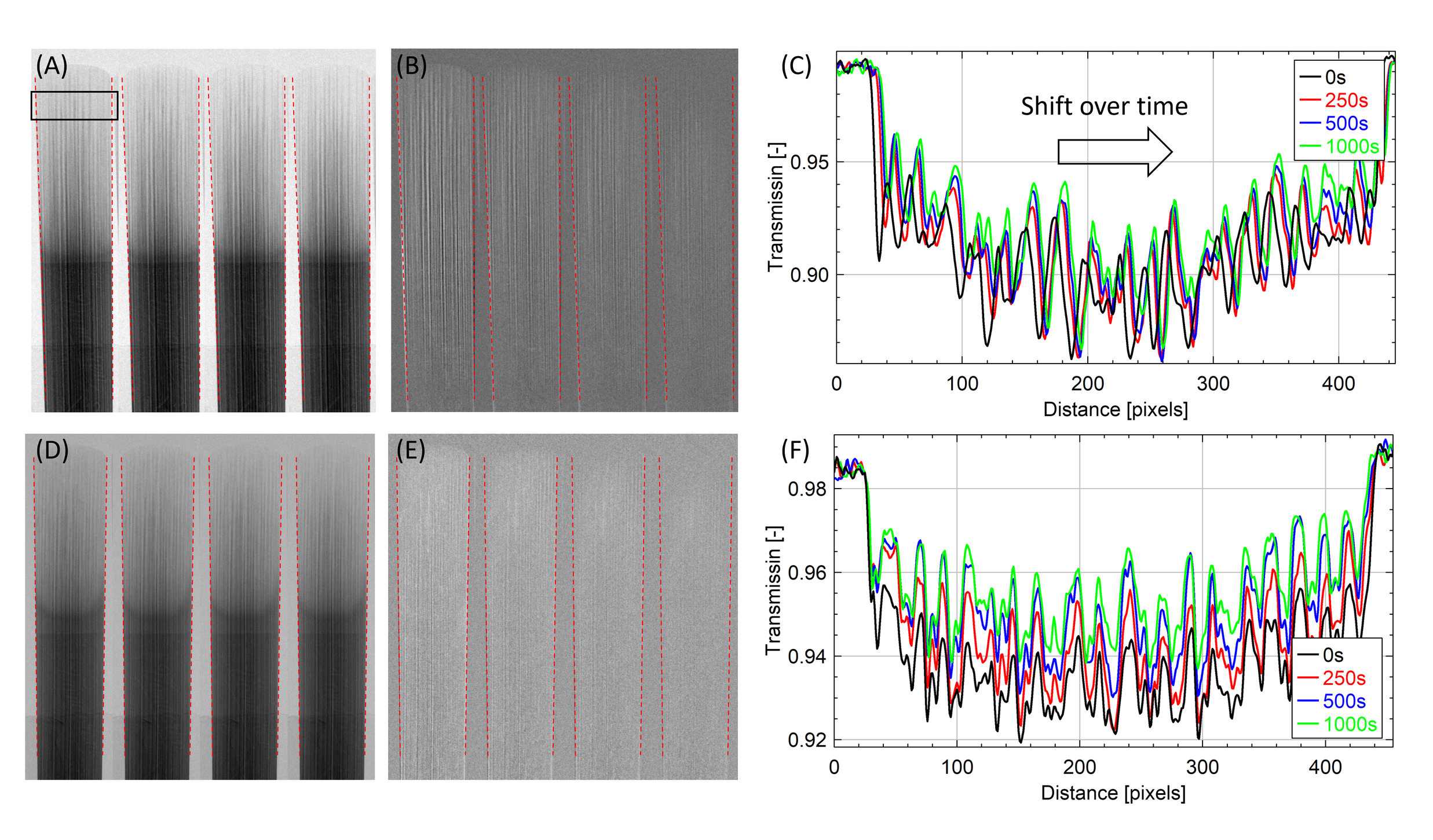

Supplement: S3 Fig — (A), (B), and (C): Transmission image (0, 250, 500, and 1000s from left to right), water thickness image, and horizontal profile corresponding to highlighted box in (A) of Reed 1 (BoL) after 2-min wetting. (D), (E), and (F): corresponding for Reed 2 (BoL) after 2-min wetting. The dotted lines in images indicate the edges of the reeds. (TIF) [file pone.0334660.s003.tif]

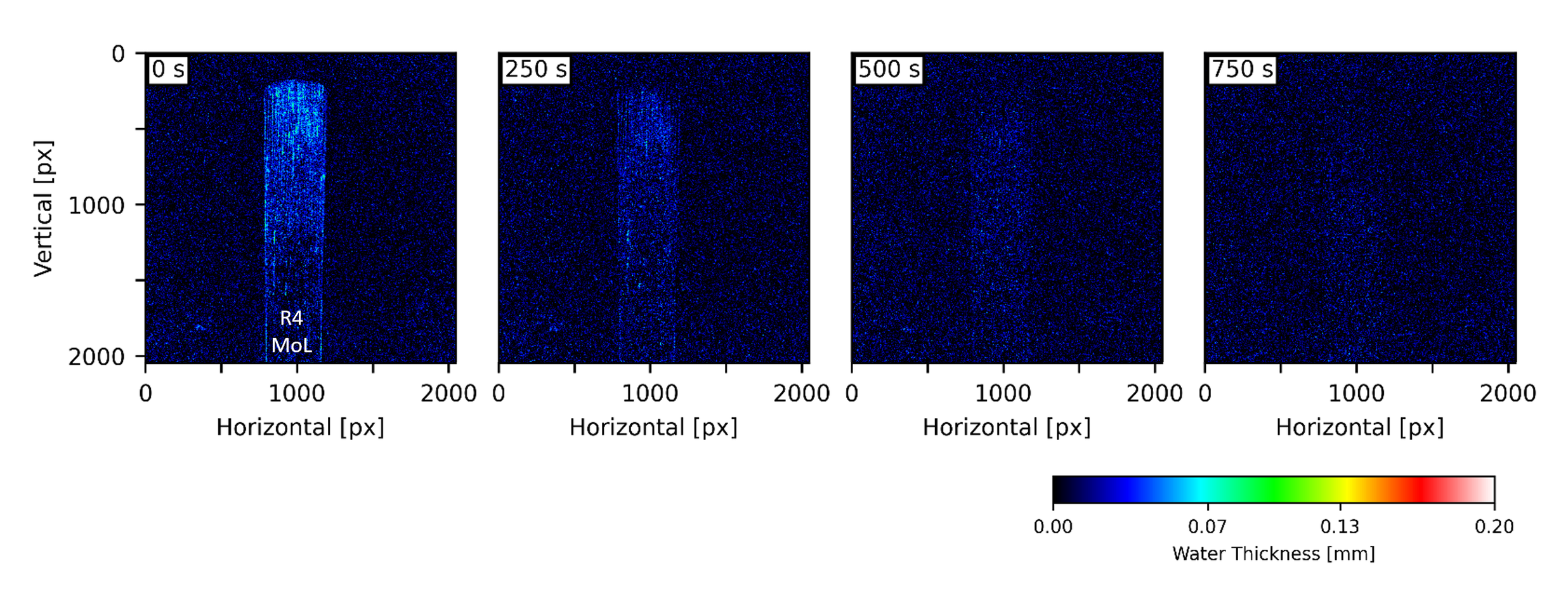

Supplement: S4 Fig — (TIF) [file pone.0334660.s004.tif]

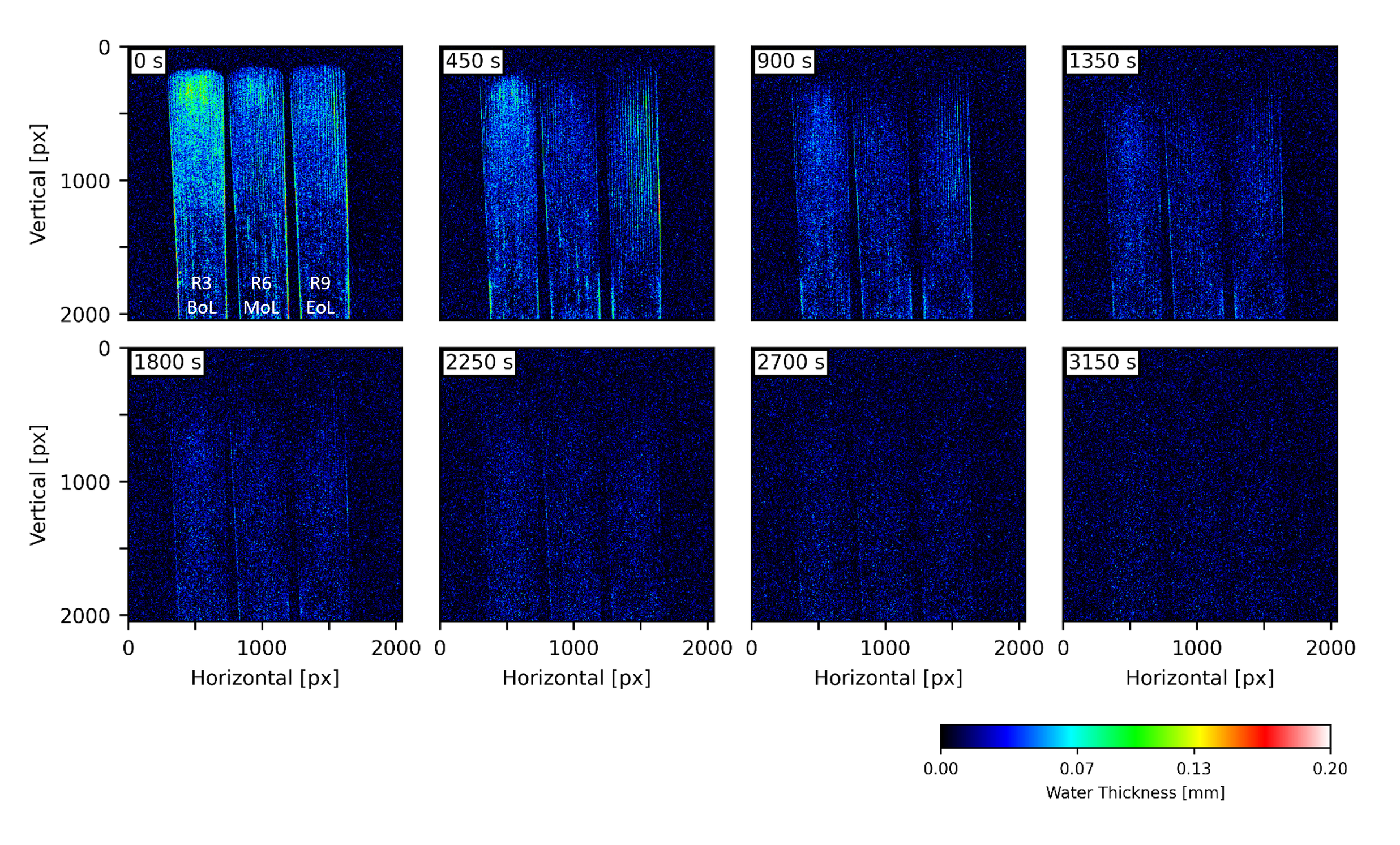

Supplement: S5 Fig — (TIF) [file pone.0334660.s005.tif]

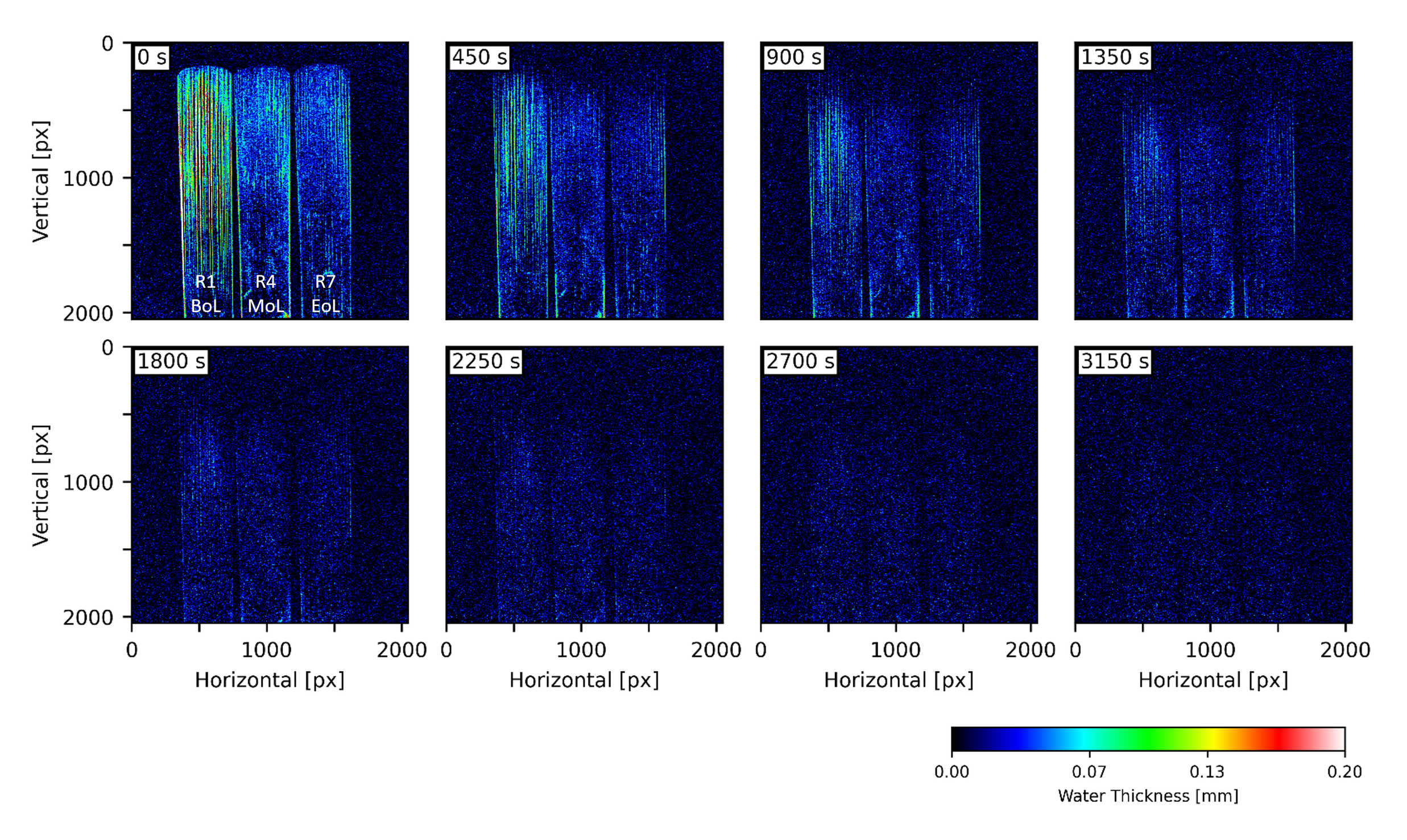

Supplement: S6 Fig — (TIF) [file pone.0334660.s006.tif]

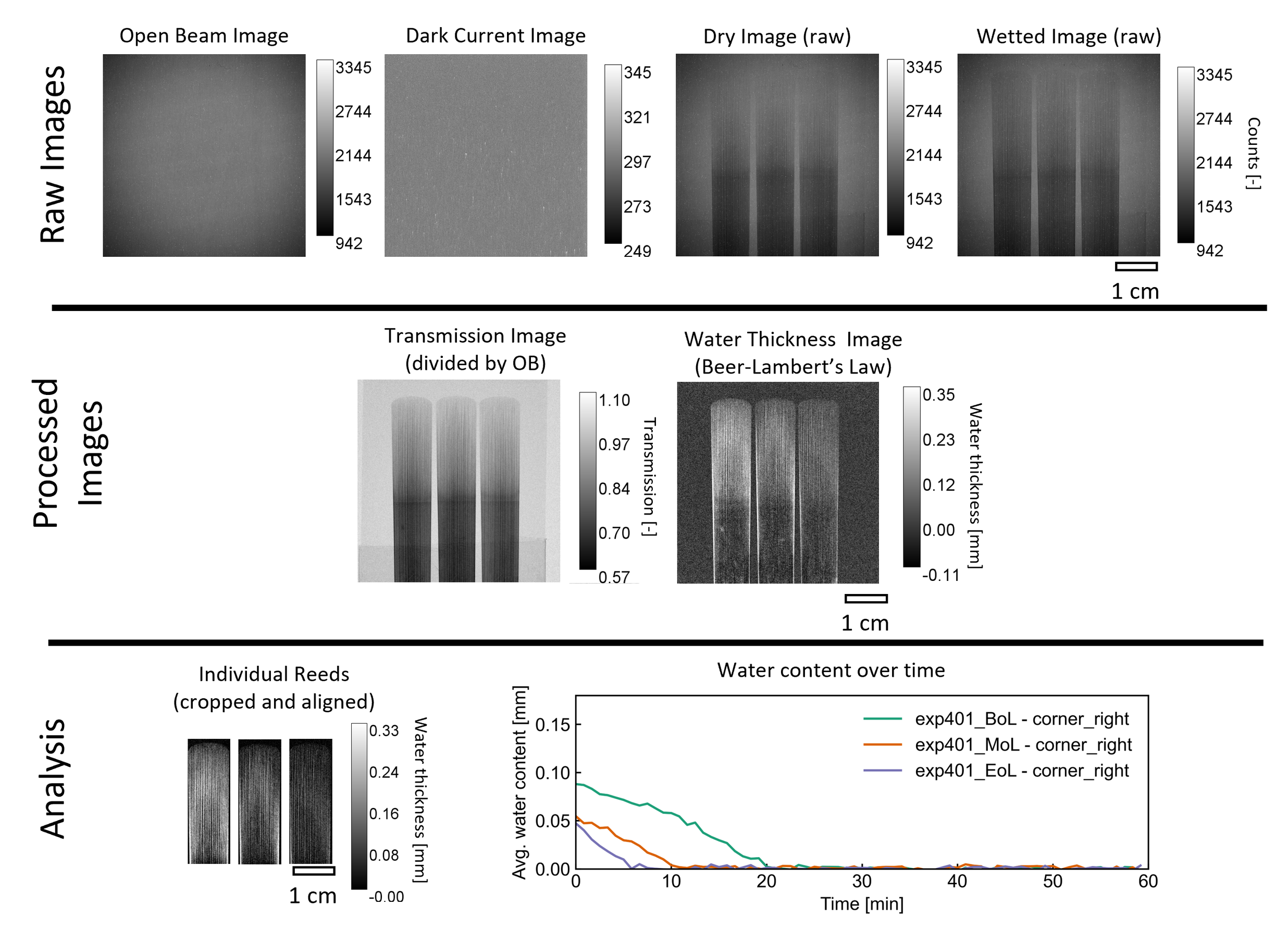

Supplement: S7 Fig — (TIF) [file pone.0334660.s007.tif]
